# Prospective multicenter real-world *RAS* mutation comparison between OncoBEAM-based liquid biopsy and tissue analysis in metastatic colorectal cancer

**DOI:** 10.1038/s41416-018-0293-5

**Published:** 2018-11-23

**Authors:** Jesús García-Foncillas, Josep Tabernero, Elena Élez, Enrique Aranda, Manuel Benavides, Carlos Camps, Eloisa Jantus-Lewintre, Rafael López, Laura Muinelo-Romay, Clara Montagut, Antonio Antón, Guillermo López, Eduardo Díaz-Rubio, Federico Rojo, Ana Vivancos

**Affiliations:** 1 Cancer Institute, University Hospital Fundación Jiménez Díaz, Autonomous University of Madrid, Madrid, Spain; 2 Medical Oncology Department, Vall d’Hebron Institute of Oncology (CIBERONC), Barcelona, Spain; 3 Medical Oncology Department, Vall d’Hebron University Hospital, Barcelona, Spain; 4 Medical Oncology Department, Reina Sofía University Hospital (UCO, IMIBIC, CIBERONC), Córdoba, Spain; 5 Medical Oncology Department, Virgen de la Victoria University Hospital, Malaga, Spain; 6 Medical Oncology Department, General University Hospital of Valencia, Valencia, Spain; 7 Department of Medicine, Universitat de Valencia (CIBERONC), Valencia, Spain; 8 Molecular Oncology Laboratory, General University Hospital Research Foundation, Valencia, Spain; 9 Department of Biotechnology, Universitat Politècnica de València (CIBERONC), Valencia, Spain; 10 Traslational Medical Oncology Group/Liquid Biopsy Analysis Unit, Health Research Institute of Santiago (IDIS), University Hospital of Santiago de Compostela (CIBERONC), Santiago, Spain; 11 Dept. of Medical Oncology, Hospital del Mar (IMIM, CIBERONC), Barcelona, Spain; 12 Medical Oncology Department, Miguel Servet University Hospital-ISS Aragon (CIBERONC), Zaragoza, Spain; 13 Department of Medical Oncology, Cruces University Hospital, Barakaldo, Spain; 14 Department of Medical Oncology, San Carlos Clinic Hospital (CIBERONC, IdISCC), Madrid, Spain; 15 Pathology Department, IIS-Fundación Jimenez Diaz (CIBERONC), Madrid, Spain; 16 Cancer Genomics Group, Vall d’Hebron Institute of Oncology, Barcelona, Spain

## Abstract

**Background:**

Liquid biopsy offers a minimally invasive alternative to tissue-based evaluation of mutational status in cancer. The goal of the present study was to evaluate the aggregate performance of OncoBEAM RAS mutation analysis in plasma of colorectal cancer (CRC) patients at 10 hospital laboratories in Spain where this technology is routinely implemented.

**Methods:**

Circulating cell-free DNA from plasma was examined for *RAS* mutations using the OncoBEAM platform at each hospital laboratory. Results were then compared to those obtained from DNA extracted from tumour tissue from the same patient.

**Results:**

The overall percentage agreement between plasma-based and tissue-based *RAS* mutation testing of the 236 participants was 89% (210/236; kappa, 0.770 (95% CI: 0.689–0.852)). Re-analysis of tissue from all discordant cases by BEAMing revealed two false negative and five false positive tumour tissue *RAS* results, with a final concordance of 92%. Plasma false negative results were found more frequently in patients with exclusive lung metastatic disease.

**Conclusions:**

In this first prospective real-world *RAS* mutation performance comparison study, a high overall agreement was observed between results obtained from plasma and tissue samples. Overall, these findings indicate that the plasma-based BEAMing assay is a viable solution for rapid delivery of *RAS* mutation status to determine mCRC patient eligibility for anti-EGFR therapy.

## Introduction

Colorectal cancer (CRC) remains one of the most common cancers worldwide, and accounts for 12% of all cancer-related deaths in Europe.^1^ The epidermal growth factor receptor (EGFR) has become an important therapeutic target in CRC,^2^ but ~40% of patients with metastatic colorectal cancer (mCRC) have tumours with mutations in *KRAS* and are not expected to respond to treatment with the anti-EGFR monoclonal antibodies cetuximab and panitumumab.^3,4^ Several studies have shown that an extended analysis of *RAS* mutations (including *KRAS* exons 2, 3, and 4 and *NRAS* exons 2, 3, and 4) may optimise the identification of patients most likely to benefit from anti-EGFR therapy,^5–9^ and clinical practice guidelines in the US and Europe include the indication for expanded *RAS* testing before the use of anti-EGFR agents.^10–12^

Typically, the evaluation of *RAS* mutation status requires the acquisition of tumour tissue, subsequent processing to formalin-fixed, paraffin-embedded (FFPE) specimens and molecular testing with various techniques. As an alternative, the analysis of circulating tumour DNA (ctDNA) can provide a rapid genotype result with a streamlined clinical workflow and minimal disturbance to the patient. The recent approval of the OncoBEAM RAS CRC liquid biopsy assay by the European Commission as an in vitro diagnostic tool allows a practical and sensible approach for determination of *RAS* mutations in ctDNA.^13^

In this study, which included 10 hospital centres across Spain certified to run OncoBEAM RAS in routine practice, we evaluated the concordance between *RAS* status determined by OncoBEAM in plasma and the reference test performed on tissue at each centre from a large cohort of mCRC patients. We also examined the characteristics of discordant cases and the mutant allele fraction (MAF) in *RAS* mutated patients.

## Patients and methods

### Study design and patients

This was a multicenter, prospective, real-world study performed in 10 Spanish centres from November 2015 to October 2016. The study was approved by the Institutional Review Board at each hospital and was conducted in accordance with the principles of the Declaration of Helsinki. Prior to participation, all patients signed the inform consent form. Newly-diagnosed mCRC patients or presenting with recurrent disease after resection and/or chemotherapy were eligible. Patients having surgery with total disease removal or that received the last cycle of chemotherapy <6 months prior to blood draw were excluded.

### Procedures

Plasma was obtained from 10 ml of blood collected in Streck cell-free DNA BCT® or EDTA tubes before any therapeutic intervention. All patients had FFPE tissue (either primary tumour or metastasis) available for mutation analysis. OncoBEAM™ RAS CRC assay, which detects 34 mutations in *KRAS/NRAS* codons 12, 13, 59, 61, 117, and 146, was used to analyse *RAS* mutations and determine MAF in plasma. The mutation profile in tissue samples was determined by standard-of-care (SoC) procedures validated by each hospital (Supplementary Table S1). Tissue *RAS* testing by BEAMing (1% mutant allele cut-off) was centrally performed by the Service Laboratory of Sysmex Inostics. The commercially available mutation testing service using the RAS OncoBEAM panel (33 single mutations, covering the same base exchanges like the IVD kit product OncoBEAM™ RAS CRC assay) was used in the laboratory of Sysmex Inostics GmbH, Hamburg (Germany) on FFPE samples from every patient case where the SoC *RAS* result was discordant with the plasma *RAS* result. The same tissue block was used for the central re-analysis by BEAMing.

### Statistical analysis

Categorical variables were summarised in numbers and percentages, continuous variables were presented as medians, minima and maxima. Concordance between plasma and tissue RAS testing was determined using a Kappa statistic (kappa) with 95% confidence interval (CI). Positive percent agreement (PPA), negative percent agreement (NPA) and overall percent agreement (OPA) were also calculated. For MAF levels correlations with clinical variables, we performed non-parametric statistics (Mann–Whitney *U* test for dichotomous and Kruskal–Wallis test for polychotomous variables). All statistical tests were considered significant when *P* < 0.05. Statistical analyses were performed using the SAS version 9.4 statistical software.

## Results

### Patient characteristics and RAS mutation status analysis from plasma and tissue

A total of 239 mCRC patients were initially included, 3 of which were excluded because total disease removal during primary surgery. The remaining 236 participants, 144 men and 92 women, comprised the study population (see their baseline characteristics in Table 1). The majority of patients (95.4%) had colorectal adenocarcinoma with distant metastases at diagnosis (85.1%), 50.4% underwent surgery to remove the primary tumour or some portion of metastasis (16.9%) before blood sample collection. The most frequent site of metastasis was the liver (71.2%) followed by the lung (29.3%).

**Table 1:** Summary of patient/tumour characteristics and mutational analysis

	Number	Percentage
Age in years		
Median (range)	65 (33–89)	—
Gender		
Male	144	61%
Female	92	39%
Time since diagnosis (days)		
Median (range)	44 (0–3971)	—
Primary site		
Right	71	30.1%
Left	162	68.6%
Not available	3	1.3%
Histological type		
Adenocarcinoma	225	95.3%
Other	11	4.7%
Number metastatic sites at ctDNA collection		
1	145	61.4%
2	74	31.4%
3+	17	7.2%
Metastatic site		
Liver (only in liver)	168 (90)	71.2% (38.1%)
Lung (only in lung)	69 (16)	29.3% (6.8%)
Peritoneal (only peritoneal)	63 (25)	26.7% (10.6%)
Other (only other)	46 (14)	19.5% (5.9%)
Tumour sample for *RAS* testing		
Primary	204	87.2%
Metastasis	30	12.8%
Plasma BEAMing result		
Mutated	121	51.3%
WT	115	48.7%
Tissue SOC result		
Mutated	131	55.5%
WT	105	44.5%
Tissue SOC + Tissue BEAMing result		
Mutated	128	54.2%
WT	108	45.8%
MAF (plasma)		
Median (range)	2.9 (0–68)	—

*MAF* Mutated allele fraction, *SOC* standard of care, *WT* wild-type

**Fig. 1:**
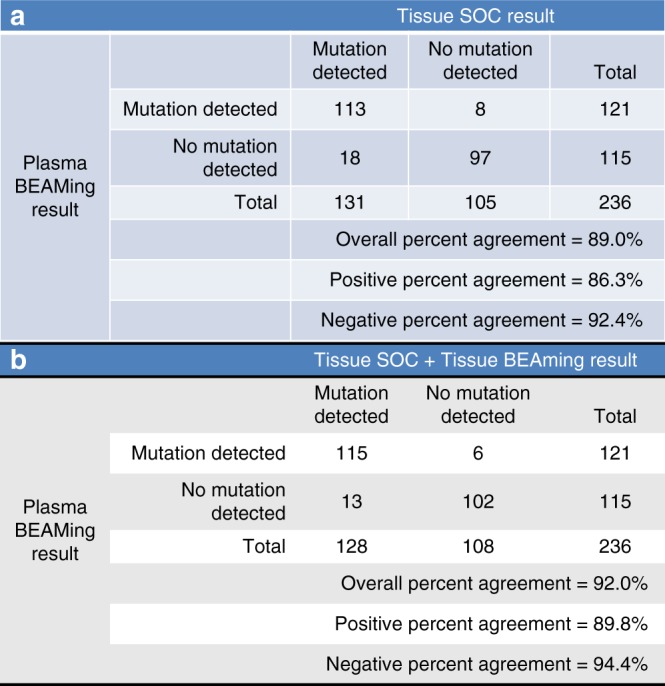
Concordance between plasma and tissue results obtained by SOC (**a**) or SOC + BEAMing (**b**). SOC standard of care

*RAS* mutation status was evaluable in both plasma and tissue of all 236 patients. Overall, *RAS* mutations were detected in 55.5% of tumour-tissue samples and in 51.3% of plasma samples (Table 1). The OPA of *RAS* results between ctDNA and SoC for tissue analysis was 89% (210/236 patients), with a kappa index of 0.770 (95% CI: 0.689–0.852) (Fig. 1a). To clarify the 26 discrepant *RAS* status results, all FFPE samples except one (not available) were centrally re-analysed by BEAMing technology (Table 2). Of the 18 plasma WT/*RAS*+ cases, five were finally concordant (Plasma WT/Tissue SoC Mutated/Tissue BEAMing WT); of the 8 plasma *RAS*+/tissue WT cases, two were concordant (Plasma BEAMing Mutated/Tissue SoC WT/Tissue BEAMing Mutated). This final analysis resulted in a total 217 concordant patients from the 236, representing a 92% overall concordance (*κ*: 0.853, 95% CI: 0.786–0.920) (Fig. 1b).

**Table 2:** Descriptive summary of discordant plasma-tissue cases (*n* = 26)

Case ID	Surgery of primary tumour?	Surgery of metastases?	Date of tissue biopsy	Date of blood collection	Plasma BEAMing result	Tissue SOC result (method)	Tissue BEAMing result	Site of metastases	Metastasis excised by surgery	Type of sample
14383	Yes	Yes	01/09/2015	28/01/2016	WT	Mutated (Pyroseq)	WT	Lung	Lung	Metastasis
14657	Yes	No	25/11/2013	03/03/2016	WT	Mutated (Pyroseq)	WT	Peritoneal, other		Primary tumour
14301	Yes	No	27/11/2013	30/03/2016	WT	Mutated (Therascreen)	WT	Lung		Primary tumour
14347	No	No	19/04/2016	06/05/2016	WT	Mutated (Pyroseq)	WT	Peritoneal, other		Primary tumour
14402	No	No	03/06/2016	30/06/2016	WT	Mutated (Pyroseq)	WT	Liver		Primary tumour
14333	No	No	28/10/2015	14/04/2016	KRAS12 0.262	WT (Pyroseq)	KRAS12 4.104%	Peritoneal		Primary tumour
14312	No	No	05/05/2016	01/06/2016	KRAS13 11.4	WT CLART− CMA Kit+ (Pyroseq)	KRAS13 16.796%	Liver, lung		Primary tumour
14365	Yes	Yes	03/05/2012	01/12/2015	WT	Mutated (Pyroseq)	KRAS12 37.709%	Lung	Lung	Primary tumour
14486	Yes	Yes	15/12/2015	03/12/2015	WT	Mutated (Pyroseq)	KRAS13 6.193%	Retroperitoneal	Lung	Metastasis
14493	Yes	Yes	05/02/2016	05/02/2016	WT	Mutated (Pyroseq)	KRAS 13 26.058%	Liver	Liver	Metastasis
14527	Yes	No	27/03/2016	03/03/2016	WT	Mutated (Pyrosequ)	KRAS12 8.469%	Peritoneal, rectum		Primary tumour
14477	No	No	01/08/2016	01/09/2016	WT	Mutated (Therascreen)	KRAS13 5.586%	Liver		Primary tumour
14335	Yes	No	19/11/2009	14/04/2016	WT	Mutated (Pyroseq)	KRAS146 28.706%	Lung, peritoneal		Primary tumour
14327	Yes	No	05/09/2013	06/06/2016	WT	Mutated (Pyroseq)	Not available	Lung		Primary tumour
14329	Yes	Yes	01/04/2016	06/06/2016	WT	Mutated (Pyroseq)	KRAS13 43.203%	Lung	Lung	Metastasis
14385	Yes	No	04/04/2014	11/07/2016	WT	Mutated (Pyroseq)	KRAS12 30.146%	Peritoneal		Primary tumour
14651	Yes	No	18/08/2016	27/09/2016	WT	Mutated (Pyroseq)	KRAS12 8.96%	Lung		Primary tumour
14410	Yes	Yes	10/05/2016	22/06/2016	WT	Mutated (Therascreen)	KRAS12 35.181%	Liver, suprarrenal, rectal	Liver	Primary tumour
14569	Yes	No	11/11/2013	16/09/2016	WT	Mutated (Idylla)	KRAS12 4.267%	Lung		Primary tumour
14669	No	No	29/09/2016	14/10/2016	WT	Mutated (Cobas)	KRAS13 1.300%	Liver		Primary tumour
14387	No	No	28/06/2016	05/09/2016	KRAS12 0.021	WT (Pyroseq)	WT	Bone		Primary tumour
14545	Yes	Yes	18/09/2015	11/07/2016	NRAS13 0.349	WT (Pyroseq)	WT	Liver, lung, lymph nodes	Liver	Primary tumour
14355	Yes	No	14/10/2015	29/02/2016	KRAS12 0.128	WT (Therascreen)	WT	Liver		Primary tumour
14717	No	No	07/11/2016	08/01/2016	KRAS13 0.047	WT (Therascreen)	WT	Liver, lung		Primary tumour
14391	Yes	No	10/12/2015	07/03/2016	KRAS13 5.32	WT (Pyroseq)	WT	Liver		Primary tumour
14646	Yes	Yes	30/03/2016	15/04/2016	KRAS12 0.038	WT (Therascreen + Pyroseq)	WT	Ovaric	Other	Primary tumour

The seven first cases were finally concordant according to plasma-tissue BEAMing paired results

*MAF* mutated allele fraction, *Pyroseq* pyrosequencing, *SOC* standard of care, *WT* wild-type

### Discordant samples description and factors affecting concordance

Among the samples with discordant *RAS* status between ctDNA and tissue BEAMing (*n* = 19), we observed that 5 of 13 Plasma WT/*RAS*+ Tissue cases involved patients that had exclusively lung metastases (Table 2). Among the group with *RAS*+ Plasma/Tissue WT discordance, the sites of metastases in these 6 patients were widely distributed, including locations such as ovary and bone.

Table 3 shows concordant/discordant paired samples according to different clinical and pathological factors. Concordance was lower (87.4% vs 95.7%; *P* = 0.033) in cases where primary tumour surgery was initially performed. Those with metastatic disease at diagnosis had a higher agreement than patients without metastasis (94.5% vs 78.1%; *P* = 0.006). A higher concordance of plasma and tissue *RAS* results was observed in patients having liver metastases (94.5–94.8%) versus those not having liver metastases (83.8%; *P* *=* 0.040), whereas the lowest concordance rate was associated with the presence of lung metastases only (68.8%).

**Table 3:** Plasma BEAMing vs tissue SOC + BEAMing concordance according to clinical and pathological variables

Variable	Concordance (%)	*P*-value	Plasma MAF Median (range)	*P* value
Age (years)				
≤65	87.6	0.824	3.3 (0–68)	0.459
>65	88		2.7 (0–51.8)	
Primary tumour resection				
No	95.7	**0.033**	6.9 (0–68)	**0.049**
Yes	87.4		1.8 (0–26.8)	
Metastasis resection				
No	93.4	**0.007**	3.9 (0–68)	0.169
Yes	79.1		1.3 (0–24.6)	
Histological type				
Adenocarcinoma	92	0.236	3.5 (0–68)	0.080
Other	81.8		0.2 (0–15.1)	
Metastases at diagnosis				
No	78.1	**0.006**	3.9 (0.2–24.7)	0.319
Yes	94.5		2.6 (0–68)	
Primary tumour location				
Left	96.2	0.483	3.9 (0–51.8)	0.351
Right	89.9		2.8 (0–68)	
Not available	100		11.7 (11.4–12)	
Number of metastases				
1	91	1.000	2 (0–68)	0.074
2	91.9		3.9 (0–35.7)	
>2	94.1		13 (0.3–51.8)	
Metastatic site				
Only liver metastasis	94.5	**0.040**	2.7 (0–68)	0.052
Liver and other sites	94.8		6.7 (0–51.8)	
Without liver metastasis	83.8		1.1 (0–26.8)	
Metastatic site				
Only lung metastasis	68.8	**0.012**	0.7 (0.1–24.7)	0.092
Lung and other sites	92.5		10 (0–51.8)	
Without lung metastasis	93.4		2.4 (0–68)	
Metastatic site				
Only peritoneal metastasis	95.8	0.738	1.3 (0.1–26.8)	0.835
Peritoneal and other sites	89.7		1.8 (0–51.8)	
Without peritoneal metastasis	91.3		3.6 (0–68)	
Source of tissue sample				
Metastasis	90	0.728	1.2 (0.1–51.8)	0.559
Primary tumour	91.7		3.5 (0–68)	

*MAF* mutated allele fraction, *SOC* standard of care

Bold values to remark a statistically significant difference

### MAF analysis in mutated plasma samples

For the 121 patients with detectable plasma *RAS* mutations, the median MAF [range] was 2.9% [0–68]. No differences in mutational load were observed in relation to age, tumour histology, metastasis at diagnosis, or primary site of disease (Table 3). The median MAF in ctDNA according to the number of metastatic sites was 2% [0–68] in those with one metastatic site, 3.9% [0–35.7] in those with two metastatic sites, and 13% [0.3–51.8] in those with three or more (*P* *=* 0.074). The median MAF for those with at least one liver metastasis was 6.7% [0–51.8], whereas the patients with metastases only in the lung showed a median of 0.7% [0.1–24.7].

## Discussion

A timely assessment of the current *RAS* mutation profile of mCRC patients provides the opportunity to deliver the most optimal therapy regimen matched to tumour molecular status.^14^ With a liquid biopsy we can determine the presence of circulating tumour cells (CTCs), cancer‐derived exosomes, and ctDNA. Despite the technological advances in identifying and characterising CTCs, there are still significant biological challenges that limit their clinical application.^15^ As ctDNA is released from primary tumours, CTCs, micrometastasis or overt metastases, this might better reflect the molecular changes that occur during disease progression.^16^ Mutations in ctDNA may be detected in blood using several techniques, such as digital PCR (dPCR) assays such as droplet-digital PCR (ddPCR) and BEAMing, or next-generation sequencing (NGS). dPCR platforms offer easy workflow and better allele-specific sensitivity and reproducibility than standard quantitative PCR, but are limited in its multiplexing capability.^17^ When multiple targets have to be analysed, NGS technology reduces the cost of screening compared to analysis with a lower throughput technology.^18^ The principles and different characteristics of these techniques have been reviewed elsewhere.^13,19^

To the best of our knowledge, this is the first prospective real-world study in which a *RAS* mutational analysis was compared between plasma OncoBEAM RAS CRC assay and tissue-based techniques in a network of hospital laboratories certified to perform OncoBEAM testing in routine clinical practice. Overall, a high concordance rate of *RAS* status was observed between plasma and tissue analysis performed by SoC procedures (89%). This rate was even better when certain tissue specimens which were seemingly mischaracterised by the local SoC technique were re-evaluated with BEAMing (92%). These results support the use of plasma testing with the OncoBEAM platform as a valuable alternative to tissue SoC to identify patients eligible for anti-EGFR therapy in routine clinical practice. Moreover, concordance rates are comparable to those obtained in previous retrospective and prospective studies,^20–22^ which corroborate the consistency of the technique among different mCRC patient populations. In fact, the frequency of *RAS* mutations in patients evaluated in this study was in agreement with the results of other groups performing expanded *RAS* analysis (plasma 51.3%; tissue 55.5%).^21–23^

Some reports have demonstrated that testing of DNA from a single colorectal tumour tissue block will wrongly assign *KRAS* wild-type status in 8–11.6% of patients.^24,25^ Thus, the sole reliance on *RAS* mutation results obtained from a primary tumour sample might misinform effective treatment of residual systemic disease, imposing significant costs both clinically and financially. This sampling bias could largely be avoided by determining the *RAS* mutational status on multiple tumour blocks, but this is neither practical nor feasible. Studies evaluating inter-tumour heterogeneity between primary tumours and metastases have also revealed mutational discordance in a significant proportion of cases^26–28^ with high levels of inter-tumour heterogeneity observed between primary tumour and matched lung metastases (32.4%).^27^ Thus, mCRC patients eligible for surgical resection can have primary tumours with a *RAS* mutation and metastases without *RAS* mutations, and vice-versa. Though there is no definitive guideline for determining which sample should be tested for *RAS* mutations, it is often the case in metastatic disease that the primary site is surgically resected while distant metastases are treated with systemic therapy.

Previous studies have shown that BEAMing is an accurate technique for the mutational analysis of archival FFPE tumour tissue.^6,8,29^ In the present study, we found seven cases in which BEAMing identified the same *RAS* mutation in tissue that was identified in plasma, contrary to the original SoC result. Differences in tissue *RAS* mutation detection capabilities ranging between 3 and 20% among diverse routine methodologies have been reported,^30–32^ possibly associated with different sensitivity thresholds.^20^ Accordingly, the agreement between plasma and tissue *RAS* testing results will likely improve when both the methods of plasma and FFPE preparation are standardised, underlying the importance of selecting a reliable laboratory for routine testing.^33^ In our population, 13 patients had mutations in tissue that could not be detected in plasma, which may be attributed to tumour heterogeneity, low ctDNA shedding or low tumour burden. In fact, five of these discordant cases had archival primary tumour mutated and excised, maybe their metastases were WT or low-shedding lesions. Other authors found similar results in patients with *RAS* mutant on tissue and WT on liquid biopsy that had recurrence of the disease after surgical resection of the primary and a lower tumour burden, with metastatic lesions often localised in the lung and lymph nodes.^34^ This finding is consistent with the significantly lower MAF found in our cohort of patients subjected to primary tumour resection. In line with this, our results also showed that the degree of *RAS* mutational concordance varied according to the metastatic site, with more discrepancies in patients with lung only metastases and a higher agreement in liver metastases; similarly, Thierry et al found higher specificity of plasma mutation analysis in patients with at least one liver metastasis,^35^ whereas Kim et al.,^27^ reported higher discordance rates when compared paired primary tumour and lung samples, so it is possible that lung metastases more frequently have a different *RAS* status than other metastatic sites. Another explanation may be that tumour budding in the metastatic lesions triggers different levels of ctDNA release. Metastatic deposits in the liver are likely highly vascularised as compared to the lungs, and this may contribute to greater levels of ctDNA released in the bloodstream.

Here, the median MAF obtained by plasma BEAMing was 2.9%, higher than in Vidal et al. (1.84%)^22^ but lower than Schmiegel et al. (6.82%).^21^ In the first study, 8 of the 59 *RAS*+ patients had received previous treatment with chemotherapy ± anti-VEGF within a month prior ctDNA blood extraction and showed significantly lower *RAS* plasma MAF as compared to treatment-naive patients, whereas our population had at least 6 treatment-free months before plasma collection. It has been reported that changes in ctDNA may occur during the course of the chemotherapy, with significant reductions in ctDNA levels observed even after the first cycle.^29^ Thus, mutational load in patients exposed to therapy may decrease in parallel to radiological response.^36,37^ Indeed, Schmiegel et al.^21^ included a cohort of stage IV newly diagnosed patients with intact primary CRC whose MAF was 6.5-fold higher (9.63%) compared with those patients who presented with recurrent disease after removal of their primary tumours (1.49%). These findings highlight the significance of determining and monitoring the MAF of *RAS*+ mCRC patients throughout the course of the disease management and the impact of any surgical procedure and/or systemic treatment on it. Moreover, based on our results, the rate of release of tumour DNA into circulation may serve as an important clinical observation to consider, as highly vascularised metastatic sites (i.e. liver) and an elevated number of metastases were associated with higher MAF values.

In conclusion, ctDNA analysis by OncoBEAM RAS CRC assay is comparable to SoC tissue testing techniques. It represents a minimally invasive method easily implemented in routine clinical practice to rapidly determine mCRC patient eligibility for anti-EGFR therapy. This technique likely avoids the potential pitfalls of selecting a targeted therapy strategy based on the molecular profile of a single lesion. A unique feature of ctDNA genotyping is its ability to evaluate the extent of an individual patient’s tumour burden, eliminating sampling issues related to tissue molecular heterogeneity and the development of mutations during the metastatic process.

## Electronic supplementary material

supplementary Table1

## Availability of data and material

All necessary data are included in the manuscript. The authors have no supplementary data to share.
