# Supplementary material for: Prospective multicenter real-world RAS mutation comparison between OncoBEAM-based liquid biopsy and tissue analysis in metastatic colorectal cancer
Source: Br J Cancer. 2018 Nov 23;119(12):1464–70. doi: 10.1038/s41416-018-0293-5 (PMC6288144; doi:10.1038/s41416-018-0293-5)
Supplement: Supplementary file 1 — supplementary Table1 [file 41416_2018_293_MOESM1_ESM.docx]

**Supplementary Table 1. Methods for standard of care tissue analysis**

| **Centre ID** | **Analysis method** |
| --- | --- |
| 01 | Pyrosequencing |
| 02 | Pyrosequencing |
| 03 | Pyrosequencing |
| 04 | Pyrosequencing, Cobas, Therascreen, others (Idylla) |
| 05 | Pyrosequencing, Cobas |
| 06 | Pyrosequencing, Cobas |
| 07 | Pyrosequencing, Therascreen |
| 08 | Pyrosequencing Therascreen |
| 09 | Therascreen, Cobas, Idylla |
| 10 | Pyrosequencing, Therascreen, CLART-CMA Kit, |
